# In Vitro Probiotic Properties of *Bifidobacterium animalis* subsp. *lactis* SF and Its Alleviating Effect on Non-Alcoholic Fatty Liver Disease

**DOI:** 10.3390/nu15061355

**Published:** 2023-03-10

**Authors:** Huihui Lv, Feiyue Tao, Lingling Peng, Shufang Chen, Zhongyue Ren, Jiahui Chen, Bo Yu, Hua Wei, Cuixiang Wan

**Affiliations:** 1 State Key Laboratory of Food Science and Technology, Nanchang University, Nanchang 330047, China; ncuspylvhuihui@163.com (H.L.); ncuspytaofeiyue@163.com (F.T.); penglingling6@163.com (L.P.); shufangchen2021@163.com (S.C.); zhongyuer666@163.com (Z.R.); chenjiahui756@163.com (J.C.); weihua114@live.cn (H.W.); 2 Jiangxi-OAI Joint Research Institute, Nanchang University, 235 Nanjing East Road, Nanchang 330047, China; yubo@ncu.edu.cn

**Keywords:** *B. lactis* SF, probiotic properties, NAFLD, lipid metabolism, gut microbes, TLR4/NF-κB pathway, P13K-Akt/AMPK pathway

## Abstract

Nonalcoholic fatty liver disease (NAFLD) is a common chronic liver disease with many influencing factors. With the increasing role of the gut–liver axis in various liver diseases, research on the prevention and treatment of NAFLD with probiotics is increasing. In the present study, a *Bifidobacterium animalis* subsp. strain, *B. lactis* SF, was isolated from the feces of healthy infants and characterized by sequencing of the 16S rDNA. A systematic probiotic evaluation was carried out, and a diet-induced mouse model was constructed to study the effect and mechanism of *B. lactis* SF on diet-induced NAFLD. Results show that *B. lactis* SF has excellent gastrointestinal fluid tolerance and intestinal colonization, and strong antibacterial and antioxidant capabilities. In vivo, *B. lactis* SF modulated intestinal flora, restored the intestinal barrier, and inhibited LPS entrance into the portal circulation, which subsequently inhibited the TLR4/NF-κB and modulated the PI3K-Akt/AMPK signaling pathway, attenuated the inflammatory response, and reduced lipid accumulation. In addition, *B. lactis* SF attenuated oxidative stress and further alleviated autophagy, resulting in an ameliorative effect on NAFLD. Therefore, our study provides a new dietary method for the treatment of NAFLD.

## 1. Introduction

Nonalcoholic fatty liver disease (NAFLD) is a clinicopathological syndrome characterized by excessive deposition of fat in hepatocytes, but without alcohol and other definite liver-damaging factors [1]. It has become the most common liver disease in clinical practice [2], and is one of the potential factors that induces liver cancer [3]. In recent years, with the increased incidence and prevalence of the disease, NAFLD has been recognized as a global public health issue. Therefore, the pathogenesis and treatment of NAFLD have become one of the research hotspots in the field of liver disease. However, its pathogenesis has not been fully elucidated. The most classic theory of the pathogenesis of NAFLD is the “two-hit” theory, in which the first hit is hepatic lipid deposition and insulin resistance and the second hit is oxidative stress [4]. However, subsequent studies over the past two decades have shown that the complexity of the pathogenesis of NAFLD far exceeds that of the “two-hit” theory. As a result, the “multiple-hit” theory has been gradually proposed, which includes genetic susceptibility, dietary factors, obesity, insulin resistance, intestinal flora disturbance, liver detoxification, chronic oxidative stress, lipid metabolic disorders, inflammatory cytokines and adipokines, and changes in immunity [5]. However, there is no clear treatment for NAFLD other than lifestyle changes such as dietary modifications, regular exercise, and weight loss [6].

Gut microbiota are strongly involved in the pathophysiology of NAFLD, and changes in the composition of gut microbiota play a crucial role in NAFLD development [7]. Typical compositional changes observed in NAFLD are accompanied by changes in Firmicute/Bacteroidete ratios and increases in Gram-negative bacteria, which can exert pro-inflammatory and metabolic effects [8]. Several studies have shown that regulating gut microbiota can improve NAFLD, for example, by supplementing probiotics to selectively stimulate the growth of anti-inflammatory bacteria and inhibit the growth of pro-inflammatory bacteria and by regulating energy metabolism [9,10]. *Lactobacillus plantarum* NA136 ameliorates NAFLD by modulating the AMPK/Nrf2 pathway to regulate fatty acid metabolism and prevent oxidative stress [11]. *Bifidobacterium* V9 ameliorated diet-induced NAFLD in rats by inhibiting oxidative stress and attenuating disturbances in lipid metabolism [12]. In addition, a randomized, double-blind, placebo-controlled human trial provided evidence that supplementation with symbiotic supplement capsules (containing seven probiotics and prebiotics) improved symptoms of hepatic steatosis and fibrosis in non-obese patients with NAFLD [13].

*Bifidobacterium* is an important member of the gut microbiota. Studies over the years have shown that *Bifidobacterium* has beneficial effects. These beneficial effects include enhancing immunity [14], improving the body’s antioxidant level [15], relieving the body’s inflammatory response [16], lowering cholesterol [17], treating gastrointestinal tract diseases, and reducing the risk of certain cancers [18]. In recent decades, the isolation and property evaluation of *Bifidobacterium* have attracted extensive attention. In the present study, a new strain of *Bifidobacterium animalis* subsp. *lactis* SF was isolated from infant feces, and its growth characteristics, stress resistance, intestinal colonization ability, antioxidant ability, and antagonistic activity against pathogens were systematically evaluated in vitro. A NAFLD mouse model was constructed to explore the effect of *B. lactis* SF on lipid metabolism and liver and intestinal damage in mice, and the involved mechanisms. This study aims to provide a new reference direction for the treatment of NAFLD.

## 2. Materials and Methods

### 2.1. B. lactis SF Strains and Culture Conditions

*B. lactis* SF was isolated from the feces of a healthy infant in Nanchang and stored in the China Center for Typical Culture Collection with the conservation number CCTCC M 2021050. *B. lactis* SF was cultured in an anaerobic incubator (Gene Science, Los Angeles, CA, USA) at 37 °C in *Bifidobacterium* BS Medium (Qingdao Hope Bio-Technology Co. Ltd., Qingdao, China). Subsequently, cells were harvested, centrifuged at 5000× *g* for 5 min at 25 °C, and washed in sterile phosphate-buffered saline (PBS).

### 2.2. Growth Experiments and Endurance Test

#### 2.2.1. Growth Experiments

The growth curves of the strains were determined according to the method of Kuerman et al. [19]. The activated strain *B. lactis* SF was inoculated in BS liquid medium at 1%. Bacterial growth was monitored by measuring the optical density at 600 nm every 2 h for 32 h in an anaerobic cabinet (36 °C ± 1 °C; 80% N_2_, 10% CO_2_, 10% H_2_). Three independent experiments were performed with triplicate samples taken at each sampling point.

#### 2.2.2. Acid and Bile Salt Tolerance Properties

The acid and bile salt tolerance tests of the strain were carried out according to Huang et al. with modification [20]. The pH of BS medium was adjusted to 2.5, 3.0, 4.0, and 5.0 with hydrochloric acid. Then, l mL of the suspension was transferred into 9 mL of the above medium and incubated anaerobically at 37 °C for 3 h. Bile salt tolerance experiments were performed in medium supplemented with 0, 0.05, 0.1, 0.2, and 0.3% bile salts (Shanghai Yuanye Bio-Technology Co., Ltd., Shanghai, China), and the viable bacteria were counted at 0 and 3 h.

#### 2.2.3. Simulated Gastrointestinal Transit

The survival of *B. lactis* SF in a simulated gastrointestinal tract (GIT) was studied in a simulation model [21]. To simulate gastrointestinal transit, we sequentially fed bacterial suspensions into gastric fluid (GJ: containing 3 g/L PBS dissolved pepsin, pH 3.0), duodenal fluid (DJ: containing 1% wt/vol bile salts, pH 8.0), and intestinal fluid (IJ: containing 0.3% wt/vol bile salts, and 0.1% wt/vol trypsin, pH 8.0). A total of 1 mL of bacterial suspension was transferred into 9 mL of GJ, and the sample was incubated anaerobically at 37 °C for 90 min. Then, cells were collected, resuspended in DJ, and incubated anaerobically at 37 °C for 20 min. Finally, cells were harvested again, suspended in IJ, and incubated anaerobically at 37 °C for 120 min. Live counts were counted before and after each step of the GIT transfer.

### 2.3. Antioxidant Ability of B. lactis SF

*B. lactis* SF was incubated anaerobically at 37 °C for 24 h and centrifuged at 5000× *g* for 10 min at 4 °C. The supernatant was collected in a sterile centrifuge tube as a cell-free supernatant. In addition, the supernatant was ultrasonicated in an ice bath for 10 min, and after microscopic observation without intact cells, the supernatant was centrifuged at 10,000× *g* for 10 min at 4 °C and collected in a sterile centrifuge tube as a cell-free extract. The bacterial precipitate was washed three times with sterile PBS and resuspended in PBS again, and the cell concentration was adjusted to 10^8^ CFU/mL and prepared as a cell suspension. The cell-free supernatant (CFS), cell supernatant (CS), cell-free extract (CFE), and positive control group (PC) were analyzed for antioxidant capacity. Vitamin C was used as the positive control and *Lactobacillus rhamnosus* GG (LGG) was used as the control strain. The antioxidant activity of *B. lactis* SF was evaluated in terms of the DPPH radical scavenging rate, hydroxyl radical scavenging rate, and total reducing capacity [22].

### 2.4. Evaluation of Sensitivity to Antibiotics

The disk diffusion method (KB method) was used to determine the antibiotic sensitivity of *B. lactis* SF [21]. Briefly, the organism was inoculated in BS medium and incubated overnight at 37 °C. In total, 100 μL of 10^7^ CFU/mL diluted culture was spread on BS agar plates, and drug-sensitive paper sheets were placed on the surfaces. Plates were incubated anaerobically at 37 °C. After incubating the above plates anaerobically for 24 h, the zone of inhibition was measured with vernier calipers.

### 2.5. Adhesive Determination of Caco-2 Cells

The intestinal cell culture Caco-2 was used in the adhesion assay [23]. Caco-2 was procured from the Cell Bank of the Chinese Academy of Sciences (Shanghai, China) and cultured in Dulbecco’s modified Eagle medium (DMEM; Solarbio, Beijing, China), 10% fetal bovine serum (FBS; Gibco, Crawley, Australia), 100 U·mL^−1^ of penicillin G, and 100 μg·mL^−1^ of streptomycin. The cell culture liquid was replaced every other day. The Caco-2 cells were diluted to approximately 1.0 × 10^5^ cells/mL. Then, 1 mL of dilution was added to a six-well plate and cultured at 37 °C in an incubator with 5% CO_2_ (Thermo Scientific, Waltham, MA, USA). After 48 h, DMEM was gently aspirated, and the cells were rinsed twice with 1 mL of PBS. *B. lactis* SF (10^8^ CFU/mL) was suspended in 1 mL of DMEM medium without double antibodies and then added to the cells. After 2 h of incubation, the sample was washed three times with PBS, and adherent bacteria were released by adding 0.5 mL of 0.25% trypsin-EDTA solution (Solarbio, Beijing, China) for 1 min. Appropriate dilutions of the resulting suspension were plated onto BS agar to quantify adherent bacteria. The number of Caco-2 adherent bacteria (CFU/cell) was equal to the number of bacteria/the number of cells [24]. In this experiment, LGG with good adhesion ability was used as a positive control [25].

### 2.6. Antimicrobial Activities of B. lactis SF

*Staphylococcus aureus* CMCC 26003, *Candida albicans* CMCC 98001, *Salmonella* typhimurium ATCC 13311, *Bacillus cereus* ATCC 14579, and *Escherichia coli* O 157: H 7 were selected as indicator microorganisms to test the antimicrobial activities of *B. lactis* SF. The antimicrobial activity was investigated using the agar diffusion method [26]. Overnight culture of the indicator microorganisms was adjusted to 10^6^ CFU/mL, and 100 μL aliquots of the bacteria were spread onto Luria–Bertani agar plates; then, 200 μL of cell-free supernatant of *B. lactis* SF was added into an Oxford cup that was placed on the surface of the agar. The diameter of the inhibition zone around the cup (including that of the Oxford cup, 7.8 mm) was measured after 24 h of incubation. The experiment was performed in triplicate.

### 2.7. Animals and Experimental Groups

Five-week-old male-specific pathogen-free C57BL/6N mice (Beijing Vital River Laboratory Animal Technology Co., Ltd., Beijing, China) were housed in Nanchang Royo Biotech Co., Ltd. (Nanchang, China) under standard conditions with a light/dark cycle of 12 h. All mice were provided with ad libitum access to food and water. All experimental procedures were in accordance with the guidelines of the Institutional Animal Care and Use Committee of Nanchang Royo Biotech Co., Ltd.

Mice were randomly assigned to three groups (ND, HFD, and SF) with eight mice in each group. The ND group was fed with a normal diet and the two other groups were fed the NAFLD model diet (40% high fat, 22% high fructose, and 2% high cholesterol model diet). The mice in the ND and HFD groups were intragastrically administered with 0.1 mL sterile of PBS, and the mice in the SF group were intragastrically administered with 0.1 mL of *B. lactis* SF (1 × 10^9^ CFU/mL) for 12 weeks. After 12 h of starvation, the mice were euthanized with ether. The mouse serum, liver, colon, ileum, and cecum contents were separately collected in sterile centrifuge tubes and stored at −80 ℃ for subsequent experiments. The plasma was placed in a refrigerator at 4 °C overnight and centrifuged at 1200× *g* for 10 min. Then, the upper layer of serum was taken for storage. During the experiment, new feed was changed daily to prevent the odor of fat oxidation from affecting the mice’s feeding, and body weight was measured every day. Lee’s index was calculated as the body weight *g*^1/3^ × 10/body length (cm).

### 2.8. H&E, Masson’s, and Oil Red O Staining

Fresh liver and colon tissues were collected and soaked in a 10% formalin tissue fixative solution. Fresh liver and colon tissues were subjected to formal in-fixing, paraffin embedding, sectioning, and hematoxylin and eosin (H&E) staining by Wuhan Servicebio Technology Co., Ltd. (Wuhan, China). Fat accumulation in liver tissue was determined via oil red O staining of liver tissue. Masson’s staining was performed to observe the degree of liver fibrosis. Oil red O staining and Masson’s staining were performed on liver tissue by Wuhan Servicebio Technology Co., Ltd. (Wuhan, China).

### 2.9. Serum- and Liver-Related Index Detection

Liver samples (100 mg) were homogenized in 900 μL of normal saline at 4 °C, and then centrifuged at 4000 rpm for 10 min at 4 °C. The contents of total cholesterol (TG), triglycerides (TC), low-density lipoprotein cholesterol (LDL-C), aspartate aminotransferase (ALT), and alanine aminotransferase (AST) in the serums and livers of mice at 12 weeks were measured using commercial assay kits (Jiancheng Bioengineering, Nanjing, China) according to the manufacturer’s instructions. The contents of superoxide dismutase (SOD), catalase (CAT), glutathione (GSH), and malondialdehyde (MDA) in liver tissues were detected using commercial test kits (Jiancheng Bioengineering, Nanjing, China) according to the manufacturer’s instructions.

### 2.10. Gene Expression Analysis

Gene expression was measured with quantitative Real-time PCR [27]. High-quality RNAs (OD260/280 and 260/230) were isolated from frozen livers and colonic tissue by using the TaKaRa RNA extraction kit (Takara, Otsu, Japan), and then a transcriptor cDNA kit (Takara, Otsu, Japan) was used for cDNA synthesis according to the manufacturer’s protocol. The PCR reaction system included 5 µL of SYBR Green Mix, 0.8 µL of primer (10 µM), and 1 µL (10 ng/µL) of cDNA plus 10 µL of ddH2O supplement. The three-step PCR reaction conditions included preheating at 95 °C for 30 s followed by 95 °C for 5 s, 59 °C for 1 min, 72 °C for 30 s for 40 cycles, annealing at 65 °C for 5 s, and an extension at 95 °C for 5 s. The mRNA levels of interferon -γ (IFN-γ), interleukin-1β (IL-1β), tumor necrosis factor-α (TNF-α), interleukin-6 (IL-6), nuclear factor kappa-B (NF-κB,), Toll-like receptor 4 (TLR4), inhibitor kappa B-α (IκB-α), sterol regulatory element binding protein-1c (SREBP-1c), acetyl CoA carboxylase (ACC), insulin receptor (InsR), insulin receptor substrate (IRS-1), phosphatidylinositol 3-kinases (Pl3K), protein kinase B (Akt), mammalian target of rapamycin (mTOR), peroxisome proliferator-activated receptor-γ (PPARγ), adenosine 5′-monophosphate (AMP)-activated protein kinase (AMPK), carnitine palmitoyltransferase-1α (CPT-1α), and CCAAT/enhancer-binding protein-α (C/EBP-α) in the liver tissues were measured using real-time PCR. The mRNA levels of the pro-inflammatory factors (IFN-γ, IL-1β, TNF-α, and IL-6), NF-κB, TLR4, IκB-α, and intestinal permeability protein-related gene zonula occludens-1 (ZO-1), Claudin-3, and Occludin in the colon tissues were measured using real-time PCR.

### 2.11. DNA Extraction from Mouse Cecal Contents and 16S rRNA Gene Sequencing

Genomic DNA was extracted from the cecal contents of five mice that were randomly selected from each group, and DNA purity and concentration were tested. The 338F: 5’-ACTCCTACGGGAGGCAGCA-3’ and 806R: 5’-GGAC-TACHVGGGTWTCTAAT-3’ universal primer sets were used to amplify the V3–V4 region of the 16S rRNA gene from the genomic DNA extracted from each sample. Both the forward and reverse 16S primers were tailed with sample-specific Illumina index sequences. The PCR amplicons were purified with Agencourt AMPure XP Beads (Beckman Coulter, Indianapolis, IN, USA) and quantified using the Qubit dsDNA HS Assay Kit and Qubit 4.0 Fluorometer (Invitrogen, Thermo Fisher Scientific, Bend, OR, USA). After the individual quantification step, amplicons were pooled in equal amounts. The constructed library was sequenced on Illumina novaseq 6000 (Illumina, Santiago, CA, USA). After sequencing, reads were processed for quality filtering, the paired reads were joined, and chimera sequences were removed. Usearch software was used to cluster the reads at 97.0% similarity level and operational taxonomic unit (OTU).

The representative sequences of OTUs were annotated with the Green genes database, and then the community composition of each sample was counted at each level. Based on Beta diversity analysis, the Bray–Curtis algorithm was used to obtain the distance matrix, and principal component analysis PCOA (PCoA) images were drawn using R language tools. We used PICRUSt2 to predict the functional abundance of a sample based on the sequence abundance of marker genes in the sample, thus predicting the pathway profile of the entire community in combination with the KEGG pathway information of the gene. We used the BugBase tool to predict microbial phenotypes from pre-computed files.

### 2.12. Enzyme Linked Immunosorbent Assay

According to the kit instructions (Shanghai C-reagent Biotechnology Co., Ltd., Shanghai, China), the double antibody sandwich method was used to determine the protein expression levels of lipopolysaccharide (LPS), NF-κB, IKB-α, IKKβ, Beclin1, and microtubule-associated protein light chain 3 (LC3-II) in serum. The content of glucose and insulin in the serum was determined using an enzyme kit (Jiancheng Bioengineering, Nanjing, China). Insulin resistance index (HOMA-IR) = fasting blood glucose (GLU) (mmol/L) × fasting insulin (INS) (mIU/L)/22.5.

### 2.13. Statistical Analysis

Data were expressed as mean ± SD. Statistical analysis was performed using SPSS19.0 software (SPSS Inc., Chicago, IL, USA) or GraphPad Prism 7.0 (GraphPad Software, Inc., San Diego, CA, USA). A paired-sample *t*-test was performed to analyze the relationships between the two groups. Multiple comparisons were evaluated using one-way or two-way ANOVA, followed by Tukey’s multiple-comparison test. Statistical significance was considered at *p* < 0.05.

## 3. Results

### 3.1. B. lactis SF Has Good Probiotic Properties 

**Figure 1:**
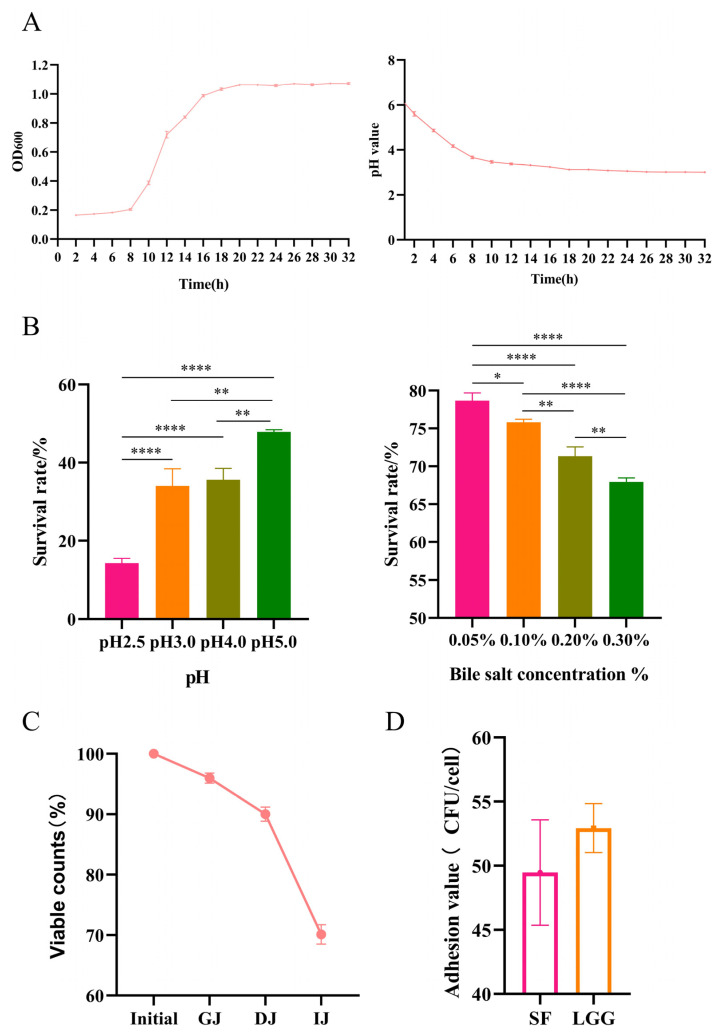

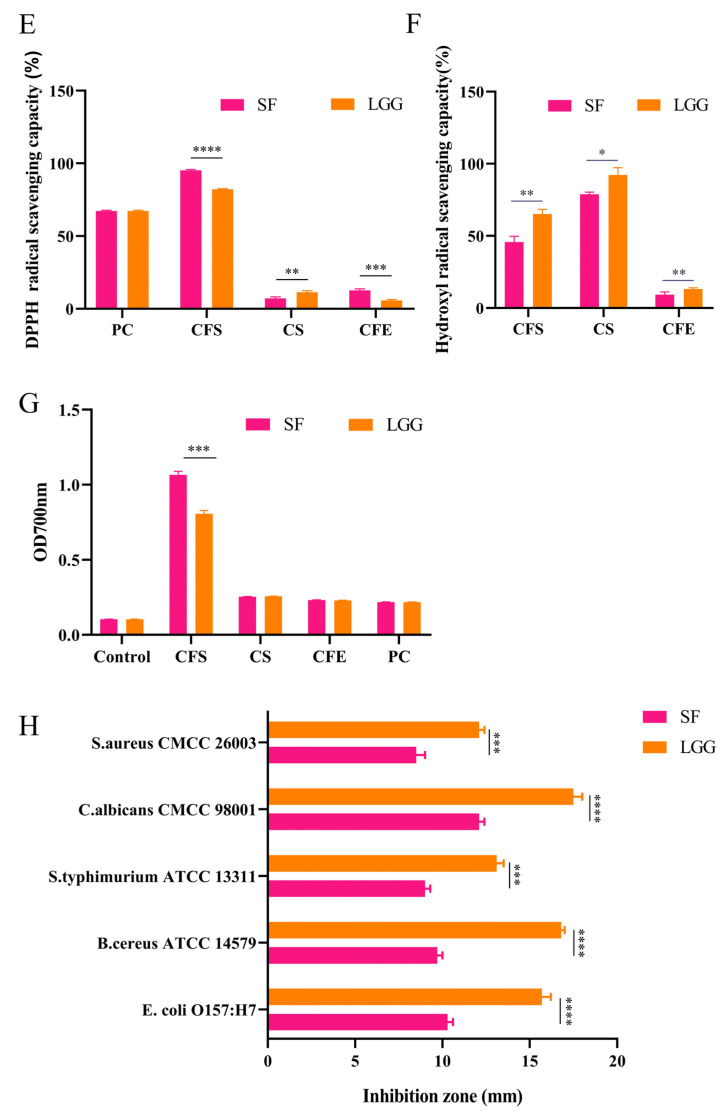
*B. lactis* SF has good probiotic properties. (**A**) Growth curve and pH change in *B. lactis* SF within 32 h. (**B**) Survival of *B. lactis* SF at different pH or different bile salt concentrations for three hours. (**C**) Survival of *B. lactis* SF in transit under the simulated Gastric-Duodenal-Intestinal tract. The percentages of survival rates were obtained at each sampling point. GJ = gastric juice; DJ = duodenal juice; and IJ = intestinal juice. (**D**) Adhesion ability of *B. lactis* SF on Caco-2 cell. (**E**) Picrylhydrazyl free radical (DPPH) radical scavenging assay (%) of *B. lactis* SF. (**F**) Hydrogen peroxide Capacity (%) on *B. lactis* SF. (**G**) Reducing power concentration on *B. lactis* SF. CFS, cell-free supernatants group; CS, cell supernatants group; CFE, cell-free extracts group; PC, Positive control group. (**H**) Antibacterial activities of the cultured supertanant of *B. lactis* SF against 5 kinds of common pathogenic bacteria. All data are presented as mean ± SD. * *p* < 0.05; ** *p* < 0.01; *** *p* < 0.001; **** *p* < 0.0001.

A strain of B. lactis SF was isolated from the feces of healthy infants and characterized by the sequencing of 16S rDNA (Sangon Biotech, Shanghai, China), and its growth and acid-producing abilities were determined. *B. lactis* SF started to grow slowly in a lag phase within 8 h and grew rapidly after 8 h. At 20 h, the highest number of cells was recorded, and the growth of the strain entered a stationary phase; *B. lactis* SF can produce organic acids, resulting in an increase in the acidity of the fermentation broth and a decrease in pH value. The pH value of *B. lactis* SF decreased gradually for 0–18 h; after 20 h, the pH value tended to be stable, and the final pH of the fermentation broth was approximately 3 (Figure 1A). It was also subjected to a first round of evaluation of survival under extreme conditions (e.g., low pH and high bile salt concentration). To ascertain the survival of *B. lactis* SF in extreme acidic conditions, a resistant test of viable cell counts at low pH was performed. Bacterial survival gradually decreased between pH 5.0 and 2.5, with 34.04% after 3 h at pH 3 and 14.30% after 3 h at pH 2.5. In addition, cell counts showed a downward trend with increasing bile salt concentrations. When the bile salt concentration was 0.30%, the bacterial survival was 67.95% (Figure 1B). Based on the study of *B. lactis* SF resistance to extreme acidity and high concentrations of bile salts, a simulated gastrointestinal transit system was further constructed, including simulated digestive enzymes, such as pepsin and pancreatin. Cell counts gradually decreased during the three stages of simulated digestion (GJ, DJ, and IJ), and the final cell survival reached 70.12% (Figure 1C). Adhesion and colonization in the digestive tract are necessary for probiotics to exert physiological functions, immune regulation, and biological antagonism [28]. LGG is a lactic acid bacterium with good adhesion and colonization properties. Therefore, in this experiment, LGG was used as a control to compare the adhesion ability of *B. lactis* SF. The results show that the adhesion index of *B. lactis* SF to Caco-2 cells reached 49.47 ± 4.11 CFU/cell, which is comparable to that of LGG (Figure 1D).

Many studies on probiotics have shown that probiotics have antioxidant functions. However, differences have been observed in the antioxidant capacities of different strains [29]. Accordingly, the antioxidant capacity of *B. lactis* SF was measured. The scavenging rates of DPPH free radicals were remarkably different among differing components of *B. lactis* SF. The CFS had a good scavenging effect on DPPH free radicals with a scavenging of 95.24%, while the scavenging of the CS and CFE were all below 20%, indicating that the active substances with DPPH free radical scavenging ability of *B. lactis* SF mainly existed in extracellular metabolites (Figure 1E). Both the CS and CFS of *B. lactis* SF showed strong hydroxyl radical scavenging ability. Among the samples, the scavenging of CFS to hydroxyl radical was 45.68%, while the scavenging of CS to hydroxyl radical was 78.77%. The scavenging of CFE to hydroxyl radicals was less than 10%, indicating that its intracellular product was weak in scavenging hydroxyl radicals (Figure 1F). The total reducing ability of *B. lactis* SF was measured. The absorbance of CFS of *B. lactis* SF reached 1.05, which is much higher than those of CS (0.25) and CFE (0.23), indicating that the reducing capacity of *B. lactis* SF fermentation supernatant far exceeded that of CSs and CFEs. Therefore, this observation suggests that the active substance in the reducing capacity of *B. lactis* SF may be an extracellular metabolic secretion (Figure 1G). The pattern of reducing power results for the positive control strain LGG was consistent with that of *B. lactis* SF (Figure 1E–G).

Antagonism against pathogenic bacteria is a prerequisite for probiotics to maintain the balance of intestinal flora and is the main probiotic property for evaluating potential probiotics [30]. The cell-free culture supernatant of *B. lactis* SF has an obvious inhibitory effect on five different common pathogens, among which *Candida albicans* CMCC 98001 had the best inhibitory effect, and the inhibition zone was as high as 12.1 ± 0.3 mm. The inhibitory effect on *Staphylococcus aureus* CMCC 26003 was weak, and the inhibition zone was 8.5 ± 0.5 mm (Figure 1H).

Considering the possibility of the combination of *B. lactis* SF and reasonable doses of antibiotics for disease treatment and its safety as a treatment method, we conducted an antibiotic susceptibility evaluation of *B. lactis* SF. *B. lactis* SF has different susceptibilities to different classes of antibiotics. It exhibits stable resistance to polypeptides (bacitracin and polymyxin B), glycopeptides (vancomycin), cephalosporins (cefoxitin), and sulfonamides (Sulphamethoxazole), while *B. lactis* SF showed stable sensitivity to sulfonamides (rifampicin), tetracyclines (tetracycline), amphenicols (chloramphenicol), and macrolides (erythromycin, Table 1). Therefore, the *B. lactis* SF screened from infant feces is a strain with excellent probiotic properties and application potential.

### 3.2. B. lactis SF Alleviated Diet-Induced Fat Accumulation and Liver Damage in Mice

**Figure 2:**
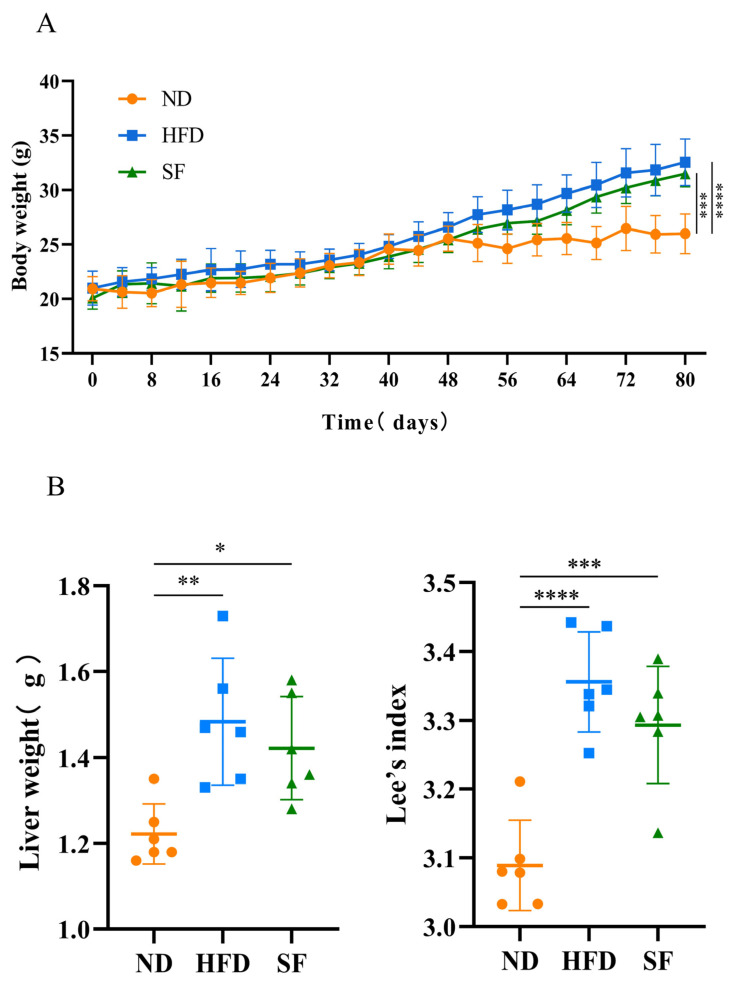

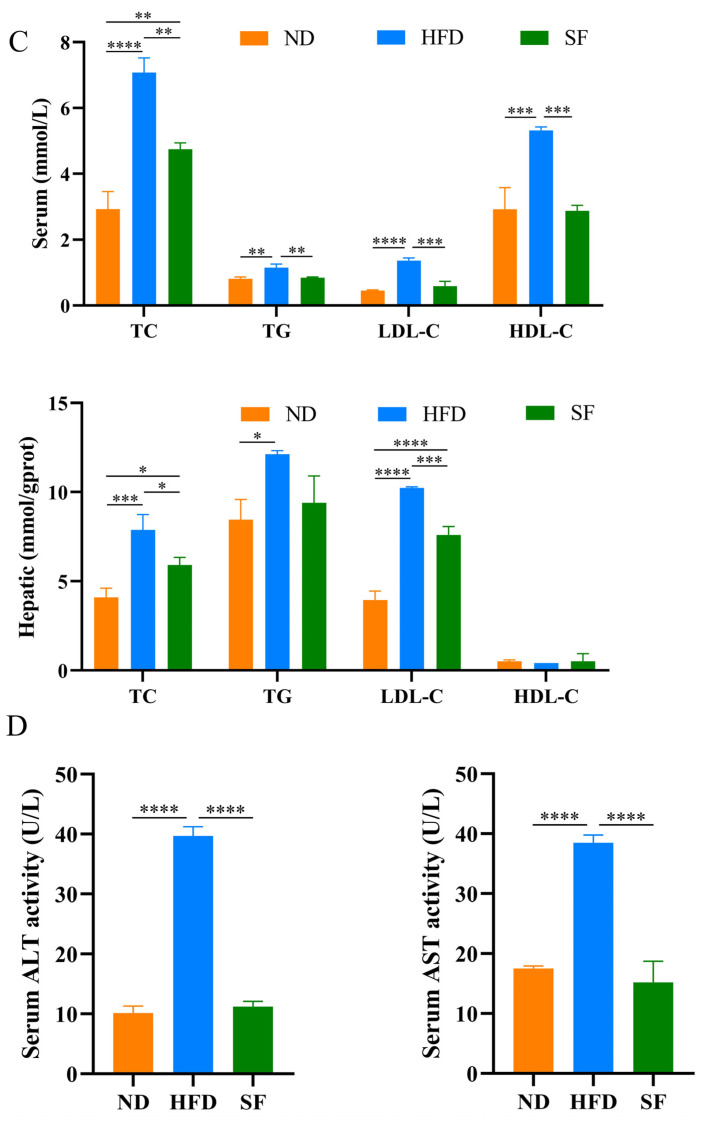

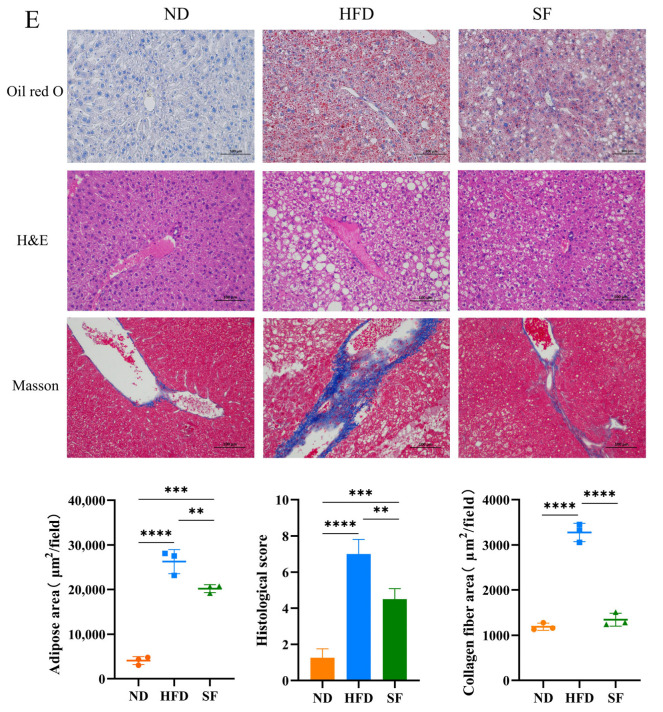
*B. lactis* SF alleviated diet-induced fat accumulation and liver damage in mice. (**A**) Changes in body weight over time in each group. (**B**) Lee’s index and liver weight (*n* = 6 mice/group; each data point represents one mouse). (**C**) Contents of total cholesterol (TG), triglycerides (TC), and low-density lipoprotein cholesterol (LDL-C) in serum and liver tissue. (**D**) Aspartate aminotransferase (ALT) and alanine aminotransferase (AST) enzyme activity in mouse serum (*n* = 3). (**E**) Oil red O staining, hematoxylin and eosin (H&E) staining, and Masson’s staining sections of mouse liver tissue and their quantification. All data are presented as mean ± SD. * *p* < 0.05; ** *p* < 0.01; *** *p* < 0.001; **** *p* < 0.0001.

Considering that a long-term high-fat, high-fructose, and high-cholesterol diet is known to cause obesity and visceral lipid accumulation [31], the body weights of the mice were recorded, and lipid accumulation in their livers was assessed. The body weights, liver weights, and Lee’s coefficients of mice in the HFD group were significantly higher than those in the ND group (*p* < 0.05), while *B. lactis* SF attenuated this increase, but not significantly (Figure 2A). In addition, the serum TCs, TGs, LDL-Cs, and liver TCs, TGs, and LDL-Cs of mice in the HFD group were significantly higher than those in the ND group. *B. lactis* SF significantly reduced the elevated levels of TC, TG, and LDL-C in serums, as well as the levels of TC and LDL-C in livers caused by the NAFLD model diet C(Figure 2B). To detect whether diet-induced liver dysfunction can be reduced by *B. lactis* SF, we analyzed the serum enzymes ALT and AST that indicate liver function. In the HFD group, the activities of ALT and AST in serum were remarkably increased. However, *B. lactis* SF substantially reduced the activities of ALT and AST (Figure 2D). The abnormal accumulation of lipids in hepatocytes is a key link in the pathogenesis of NAFLD. To explore the accumulation of lipids in hepatocytes, the oil red O staining method was used to measure the fat content in liver tissue [32]. As shown by oil red O staining, a large number of lipid droplets were accumulated in the hepatocytes of the HFD-group mice, while *B. lactis* SF could remarkably improve this phenomenon with a significant reduction in lipid droplets. Liver tissue with HE and Masson’s staining was also adopted to determine histological changes in different groups [33]. The hepatic lobule structure of the ND group was clearly discernible, the size of the hepatocytes was basically the same, the nucleus was intact, and the cytoplasm was red-stained. In the HFD group, the hepatocyte volume increased, the nucleus was slightly atrophied, fat vacuoles were common, and the infiltration of inflammatory cells was obvious. However, liver injury was significantly reduced in the SF group, and the mice showed significantly less hepatic lipid accumulation and significantly less inflammatory infiltration after 12 weeks of *B. lactis* SF administration (Figure 2E). Therefore, *B. lactis* SF could reduce fat accumulation and alleviate liver injury induced by the NFALD model diet [34].

### 3.3. B. lactis SF Reduced Diet-Induced Lipid Synthesis and Metabolism and Insulin Resistance by Modulating the P13K-Akt/AMPK Signaling Pathway

**Figure 3:**
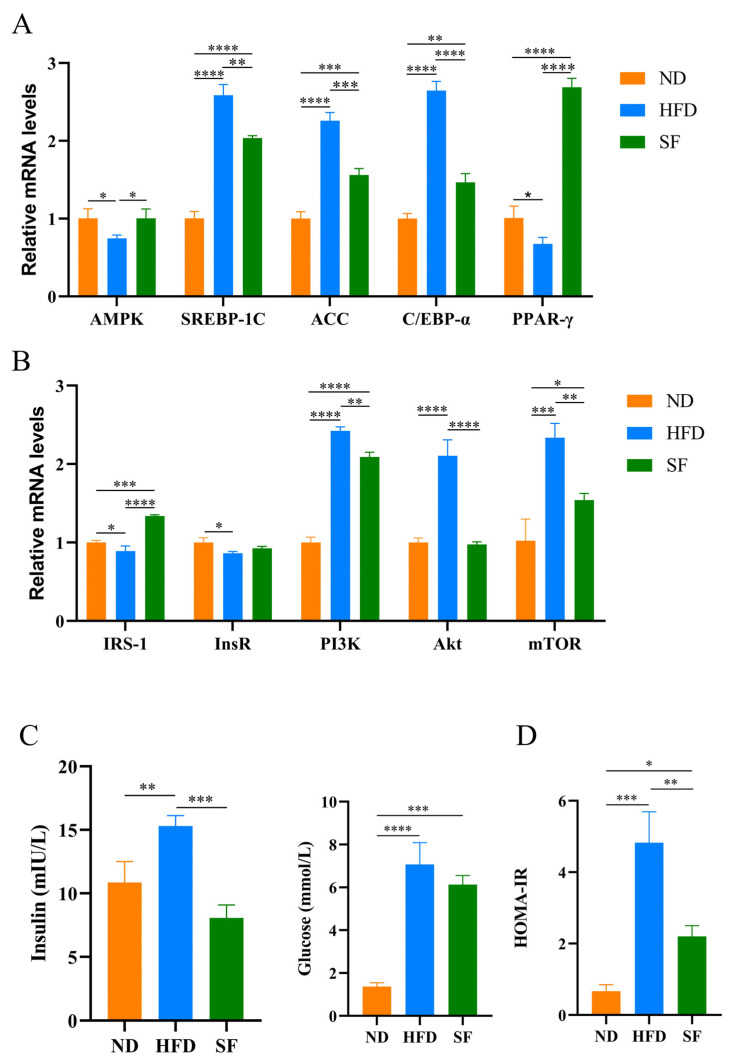
*B. lactis* SF reduced diet-induced lipid synthesis and metabolism and insulin resistance by modulating the P13K-Akt/AMPK signaling pathway. (**A**) mRNA levels of adenosine 5‘-monophosphate (AMP)-activated protein kinase (AMPK), sterol regulatory element binding protein-1c (SREBP-1c), acetyl CoA carbox-ylase (ACC), CCAAT/enhancer-binding protein-α (C/EBP-α), and peroxisome proliferator-activated receptor-γ (PPARγ) in livers (*n* = 3). (**B**) mRNA levels of insulin receptor substrate (IRS-1), insulin receptor (InsR), phosphatidyl-inositol 3-kinases (Pl3K), protein kinase B (Akt), and mammalian target of rapamycin (mTOR) in livers. (*n* = 3) (**C**) Fasting insulin and glucose levels in mice (*n* = 3). (**D**) Results of HOMA-IR in mice (*n* = 3). All data are presented as mean ± SD. * *p* < 0.05; ** *p* < 0.01; *** *p* < 0.001; **** *p* < 0.0001.

Lipid metabolism is closely related to the pathogenesis of liver disease [35]. The mRNA expression levels of some key genes in liver lipid synthesis and fatty acid oxidation were detected by qRT-PCR [36]. AMPK and PPAR-γ are key proteins in fatty acid β-oxidation. In the livers of HFD mice, the mRNA expression levels of AMPK and PPAR-γ remarkably decreased. *B. lactis* SF restored the AMPK mRNA expression to normal levels, while the mRNA expression of PPAR-γ remarkably increased in the SF group. SREBP-1c is a major regulator of fatty acid synthesis in the liver. It can regulate enzymes or proteins related to fatty acid synthesis such as ACC [37]. In the HFD group, the mRNA expression levels of SREBP-1c and ACC in the livers of mice were remarkably increased, while the mRNA expression levels of SREBP-1c and ACC were substantially decreased by *B. lactis* SF (Figure 3). C/EBPα was highly expressed in the livers of HFD mice, while *B. lactis* SF remarkably reduced C/EBPα overexpression induced by diet (Figure 3A). The expression levels of related genes involved in the mTOR pathway were also examined. Results show that the NAFLD model diet led to a significant decrease in the mRNA expression of InsR and IRS-1, whereas the liver mRNA expression of IRS-1 of mice in the SF group significantly increased. The mRNA expression of InsR also increased compared to the HFD group, but the change was not significant. In the HFD group, the mRNA expression of Pl3K, AKT, and mTOR significantly increased, while *B. lactis* SF significantly alleviated the overexpression of Pl3K, AKT, and mTOR (Figure 3B). NAFLD is often associated with insulin resistance, and fasting blood glucose and fasting insulin levels in mice were measured. The SF group reversed the diet-induced increase in fasting insulin levels and alleviated the increase in fasting blood glucose in mice (Figure 3C). The HOMA-IR index is an important measure for the evaluation of the body’s resistance to insulin. The HOMA-IR index of mice in each group was calculated and analyzed. Results showed that compared with the ND group, the HOMA-IR index of the mice in the HFD group increased, whereas *B. lactis* SF significantly reduced insulin resistance induced by diet in mice (Figure 3D). In summary, *B. lactis* SF alleviates abnormal fatty acid synthesis gene expression and insulin resistance in the livers of NAFLD mice induced by diet through regulating mRNA expression of genes associated with the P13K-Akt/AMPK signaling pathway.

### 3.4. B. lactis SF Alleviated Diet-Induced Hepatic Oxidative Stress, Inflammation, and Autophagy

**Figure 4:**
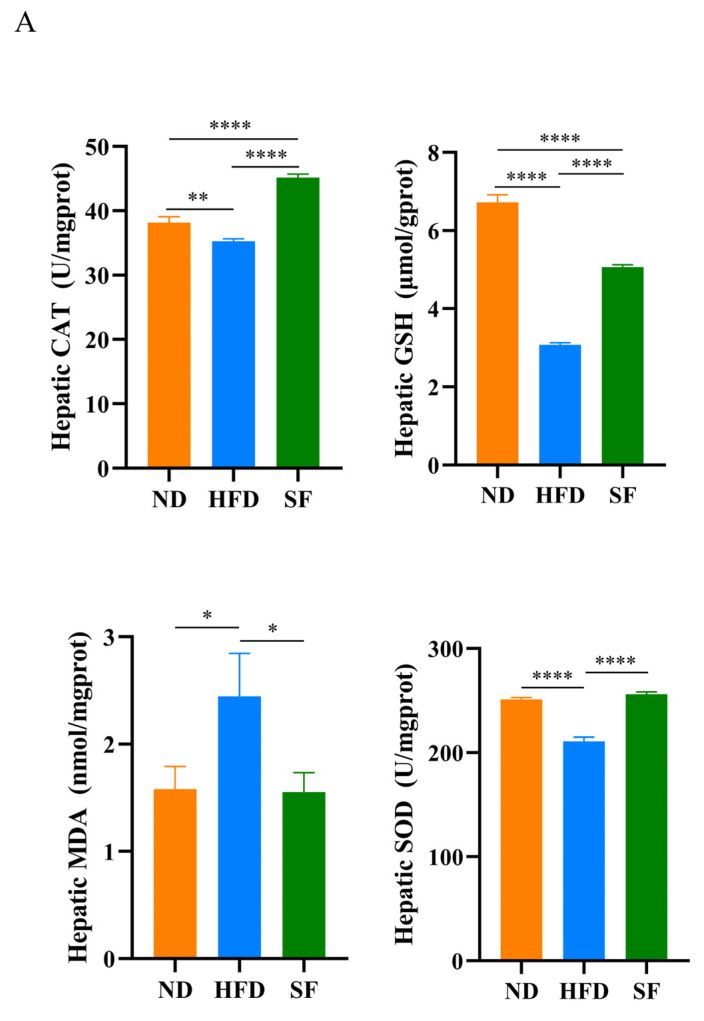

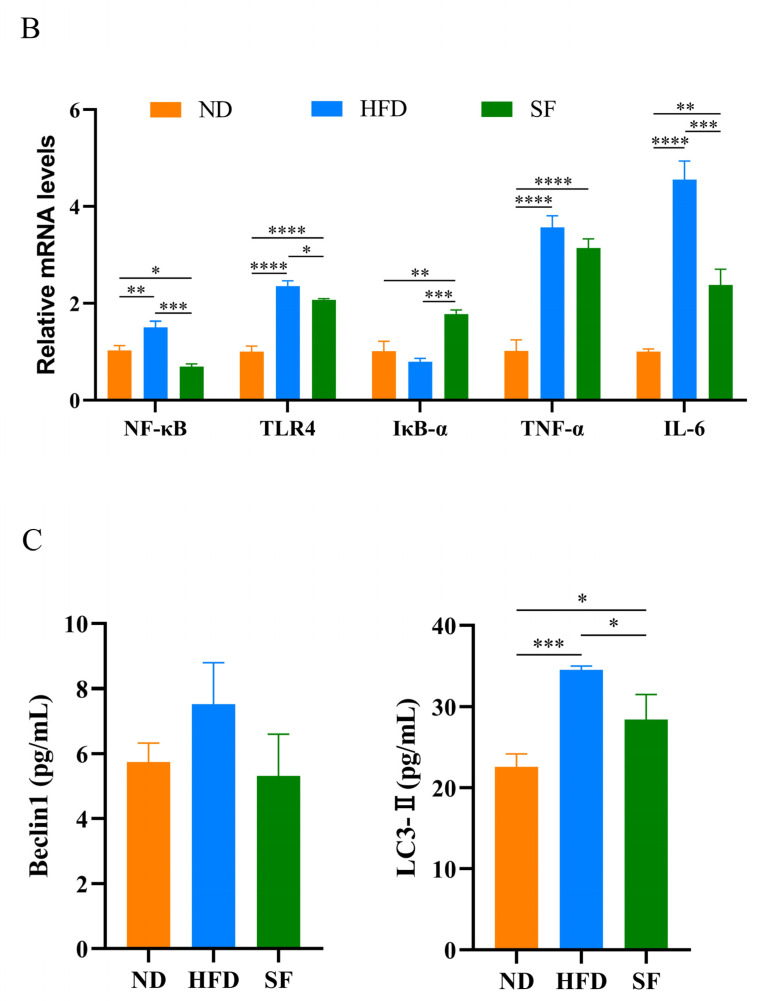
*B. lactis* SF alleviated diet-induced hepatic oxidative stress, inflammation, and autophagy. (**A**) The activities of superoxide dismutase (SOD), catalase (CAT), and the content of glutathione (GSH), and malondialde-hyde (MDA) in livers (*n* = 3). (**B**) mRNA levels of nuclear factor kappa-B (NF-κB), Toll-like receptor 4 (TLR4), inhibitor kappa B-α (IκB-α), tumor necrosis factor-α(TNF-α), and interleukin-6 (IL-6) in livers (*n* = 3). (**C**) Quantitative analysis of protein expressions of microtubule-associated protein light chain 3II (LC3-II) and beclin1 in serum (*n* = 3). All data are presented as mean ± SD. * *p* < 0.05; ** *p* < 0.01; *** *p* < 0.001; **** *p* < 0.0001.

Oxidative stress has been shown to play an important role in NAFLD pathogenesis [38]. Therefore, key oxidation indicators were investigated to explore the mechanism of action of *B. lactis* SF in alleviating NAFLD. The results showed that the NAFLD model diet significantly decreased the activities of SOD and CAT in the livers of mice, decreased the content of GSH, and significantly increased the content of MDA. By contrast, *B. lactis* SF intervention significantly increased SOD, CAT activity, and GSH content and decreased MDA content (Figure 4A). To investigate whether *B. lactis* SF can alleviate liver inflammation, we detected the expression levels of inflammation-related cytokines NF-κB, TLR4, IκB-α, TNF-α, and IL-6 in liver tissue. In comparison with the HFD group, *B. lactis* SF can reduce the mRNA expression levels of NF-κB, TLR4, TNF-α, and IL-6 in the livers of mice, and IκB-α expression levels were upregulated in the SF group (Figure 4B). The expression of autophagy-related proteins in serum were also detected. The HFD group showed higher expression levels of Beclin1 and LC3-II than the ND group. In contrast to the HFD group, the SF groups showed lower expression levels of Beclin1 and LC3-II (Figure 4C). This suggests that *B. lactis* SF can attenuate oxidative stress, inflammation, and autophagy in NAFLD mice by normalizing excessive autophagy that is brought on by NAFLD.

### 3.5. B. lactis SF Alleviated Diet-Induced Intestinal Barrier Disruption and Intestinal Inflammation by Inhibiting the TLR4/NF-κB Signaling Pathway

**Figure 5:**
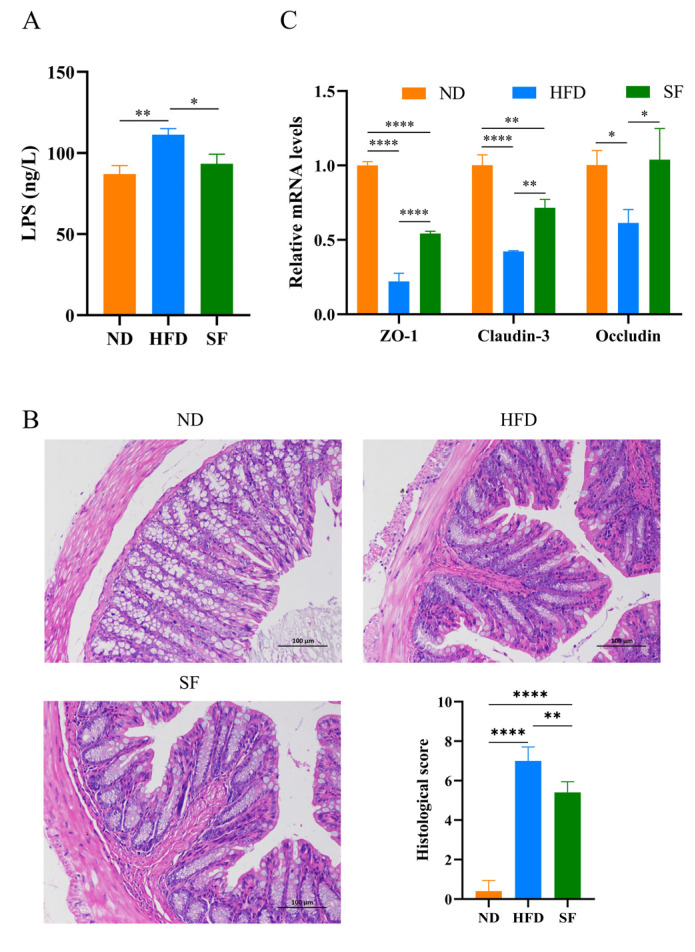

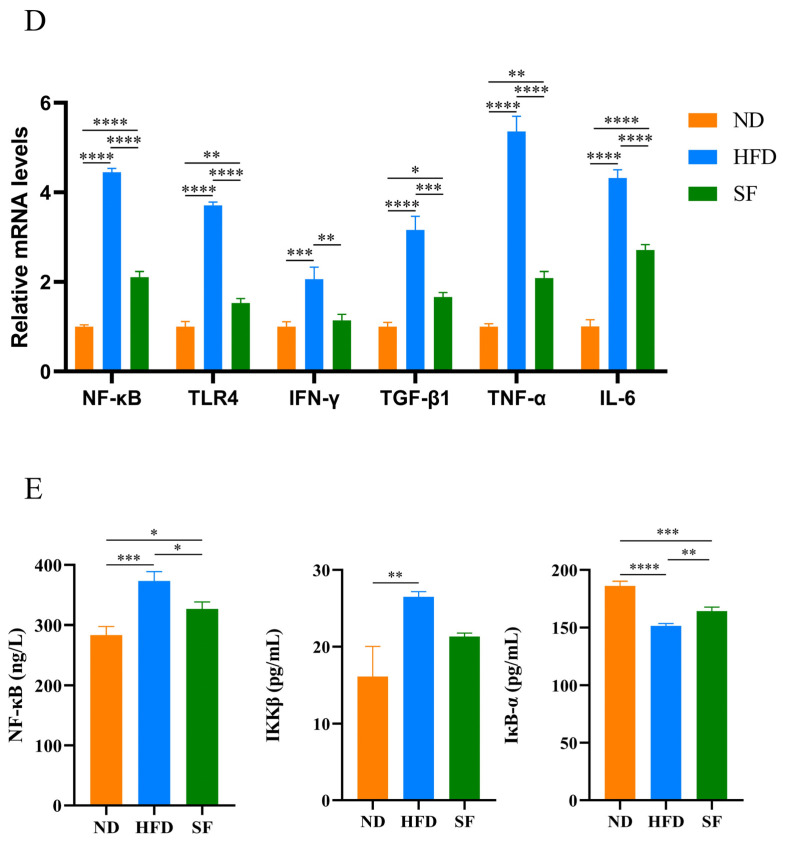
*B. lactis* SF alleviated diet-induced intestinal barrier disruption and intestinal inflammation by inhibiting the TLR4/NF-κB signaling pathway. (**A**) Lipopolysaccharide (LPS) concentrations in portal vein blood of mice (*n* = 3). (**B**) Hematoxylin and eosin (H&E) pathological section and histopathological scoring of mouse colon tissue. (**C**) Expression of tight junction proteins (Zonula occludin-1, Occludin and Claudin-3) mRNA in the intestinal tracts of mice (*n* = 3). (**D**) Nuclear factor kappa-B (NF-κB), toll-like receptor 4 (TLR4), interferon-γ (IFN-γ), transforming growth factor β1 (TGF-β1), tumor necrosis factor-α(TNF-α), and interleukin-6 (IL-6) mRNA expression in the intestinal tracts of mice (*n* = 3). (**E**) Quantitative analysis of protein expressions of NF-κB signaling in serum (*n* = 3). All data are presented as mean ± SD. * *p* < 0.05; ** *p* < 0.01; *** *p* < 0.001; **** *p* < 0.0001.

In comparison with the control mice, the HFD mice had significantly higher portal vein serum lipopolysaccharide (LPS) concentrations, suggesting that the NAFLD model diet can impair intestinal barrier function. By contrast, *B. lactis* SF significantly decreased LPS concentrations (Figure 5A). To detect the pathological damage of colon tissue, we performed HE staining [39]. Results showed that the colonic mucosas of the mice in the ND group were intact, and no inflammatory cell infiltration was observed. The colonic mucosas of the HFD group were obviously shed, the acini were destroyed, and a large number of inflammatory cells were infiltrated. The SF group had milder mucosal defects and fewer inflammatory cells (Figure 5B). Tight junctions (TJs) in the gut are involved in the integrity of the gut barrier. The expression levels of claudin-3, occludin, and zonula occludin-1 were downregulated in the HFD group and significantly increased in the SF group compared to the HFD group (Figure 5C). The mRNA expression of inflammatory factors in intestinal tissue were further detected. NF-κB, TLR4, IFN-γ, TGF-β1, TNF-α, and IL-6 expression levels in the HFD group remarkably increased, whereas *B. lactis* SF significantly decreased the expression of these factors (Figure 5D). The expression of NF-κB signaling-related proteins in serum was also examined. The concentrations of IKK-βand NF-κB were significantly increased in the HFD group, while those of IκB-α were significantly reduced. However, in the SF group, both IKK-β and NF-κB decreased, but IκB-α increased significantly (Figure 5E). The TLR4/NF-κB signaling pathway was activated, while *B. lactis* SF inhibited the activation of this signaling pathway and the release of pro-inflammatory factors. In conclusion, *B. lactis* SF can reduce intestinal inflammation and restore the intestinal barrier by inhibiting the TLR4/NF-κB signaling pathway.

### 3.6. B. lactis SF Attenuated Diet-Induced Intestinal Dysbiosis in Mice

**Figure 6:**
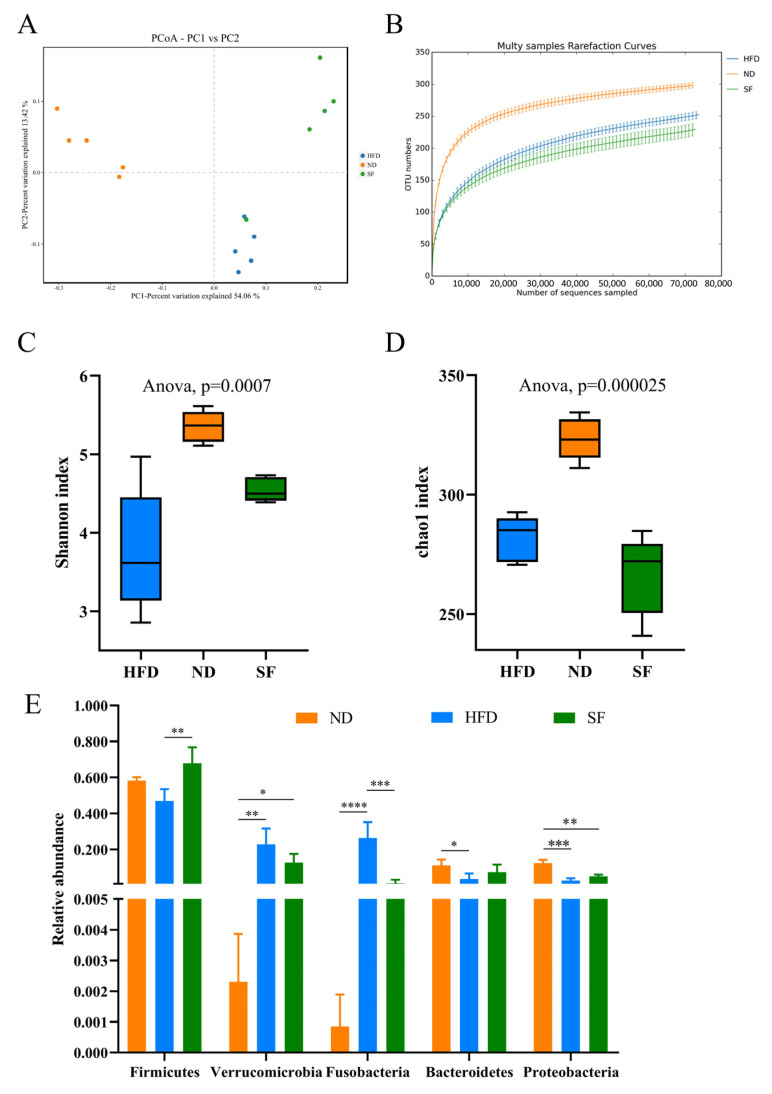

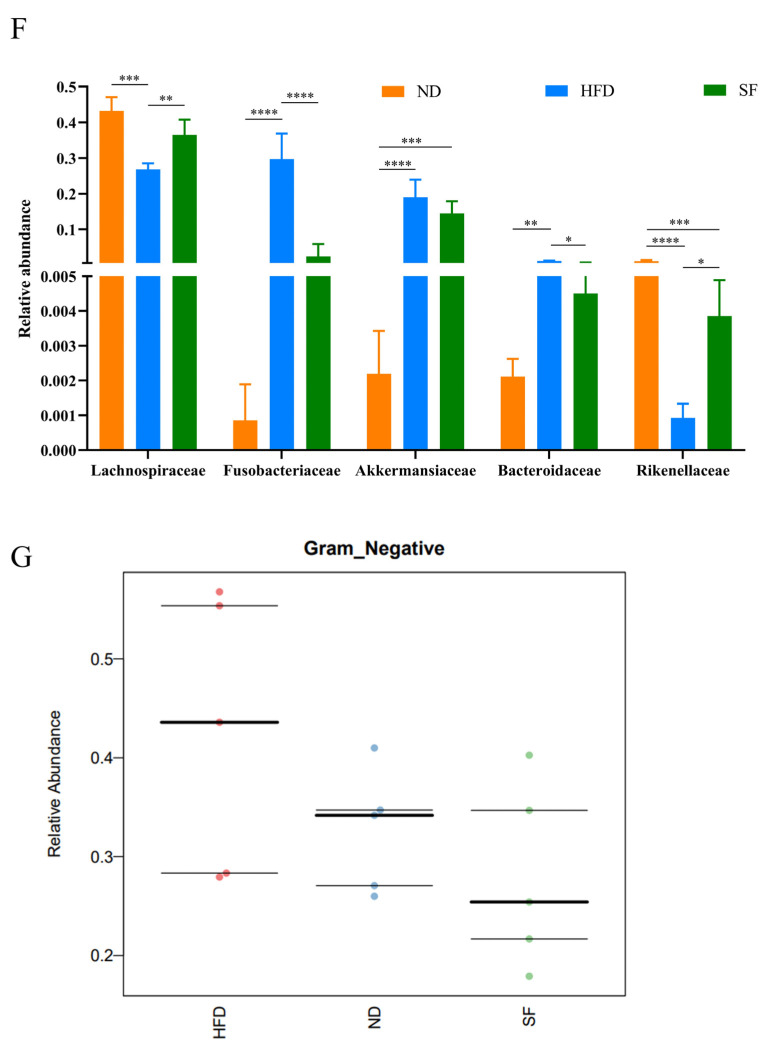

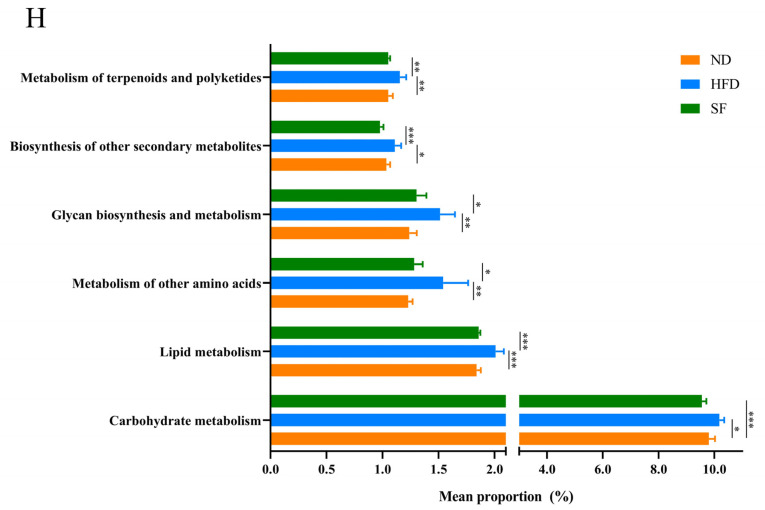
*B. lactis* SF attenuated diet-induced intestinal dysbiosis in mice. (**A**) Rarefaction curve (*n* = 5). (**B**) PCoA analysis chart, PC1 vs. PC2: the dots represent each sample and different colors represent different groups, and the horizontal and vertical coordinates are the two characteristic values that cause the largest differences between the samples with the main influence degree reflected in the form of percentage (*n* = 5). (**C**) Shannon index (*n* = 5). (**D**) Chao index (*n* = 5). (**E**) Analysis of Variance between groups at the phylum level (*n* = 3–5). (**F**) Analysis of Variance between groups at the family level (*n* = 3–5). (**G**) Relative abundance of Gram-negative bacteria obtained by BugBase. (**H**) Relative abundance percentage of metabolic pathways of intestinal flora (*n* = 5). All data are presented as mean ± SD. * *p* < 0.05; ** *p* < 0.01; *** *p* < 0.001; **** *p* < 0.0001.

Given the close connection between the microflora and the liver, changes in the microbial composition were investigated to further uncover the hepatoprotective effect of *B. lactis* SF. To assess the differences in gut microbiota diversity between groups, we analyzed the alpha and beta diversity. Significant differences in gut microbiota composition were observed among ND, HFD, and SF mice through weighted Unifrac PCoA (Figure 6A). Moreover, the rarefaction curves tended to be relatively flat, illustrating that the sequencing quantity and depth complied with the requirement for subsequent analysis (Figure 6B). The alpha diversity of the intestinal microbial population was reflected by the diversity index (Shannon) and community abundance index (Chao). In comparison with the control group, the NAFLD model diet significantly decreased the Shannon and Chao indices; the Shannon index in the SF group was higher than that in the HFD group (Figure 6C), and the Chao index was lower than that in the HFD group (Figure 6D). Therefore, the NAFLD model diet and *B. lactis* SF intervention significantly altered the richness and diversity of gut microbes in mice.

The changes in the cecum microbiota composition at the phylum and family levels were further analyzed. At the phylum level (Figure 6E), Metastats analysis showed that the NAFLD model diet decreased the abundance of Firmicutes, Bacteroidetes, and Proteobacteria, while increasing *Verrucomicrobia* and *Fusobacterium*; however, *B. lactis* SF prevented these changes (Figure 6E,G). Metastats analysis was further used to compare the differences in family levels among these groups. At the family level (Figure 6F), the relative abundances of *Fusobacteriaceae* and *Bacteroidaceae* in the HFD group were higher than those in the ND group, while those of *Lachnospiraceae* and *Rikenellaceae* were significantly lower than those of the two other groups. In comparison with the ND group, the relative abundance of *Akkermansiaceae* was significantly increased in the HFD and SF group.

In addition, the BugBase phenotype prediction results showed that the relative abundance of Gram-negative bacteria in the gut microbiota of mice in the HFD group increased, but this value was decreased by *B. lactis* SF treatment (Figure 6G). KEGG analysis predicted by PICRUSt showed that the gut microbiota of HFD mice enhanced the functions in bacterial carbohydrate metabolism, lipid metabolism, amino acid metabolism, glycan biosynthesis and metabolism, biosynthesis of other secondary metabolites, and metabolism of terpenoids and polyketides, and similar levels were observed between the SF and ND groups (Figure 6H).

## 4. Discussion

NAFLD is a common chronic liver disease that is closely associated with metabolic syndrome and insulin resistance. Multiple risk factors are involved in the pathogenesis of NAFLD, including genetic and dietary factors, oxidative stress, the distribution of adipose tissue, and dysbiosis of the gut microbiota [40]. With the determination of the important role of the gut–liver axis, which is closely related to the occurrence and development of NAFLD, more studies have been conducted on the treatment of NAFLD with probiotics. We screened out a strain of *B. lactis* SF from infant feces. The probiotic properties were systematically evaluated in vitro and showed good growth characteristics, stress resistance, intestinal colonization, and antioxidant and pathogenic antagonism. Therefore, we investigated the effects of *B. lactis* SF and explored its mechanism of action by constructing a mouse model of NAFLD caused by diet.

A high-fat diet can lead to a disturbance of intestinal flora in the body, which is crucial to the occurrence and development of NAFLD [41]. Intestinal microbiota disturbances are often manifested in decreased bacterial diversity and changes in structural composition. The relative abundance of *Bacteroidetes* in gut microbiota is correlated with liver health, and the *Firmicutes*/*Bacteroidetes* ratio can indicate the host’s energy metabolism [42]. Results showed that the NAFLD model diet altered the intestinal microbiota structure in mice, resulting in intestinal dysbiosis, while *B. lactis* SF alleviated diet-induced gut microbiota dysbiosis by adjusting the *Firmicutes*/*Bacteroidetes* ratio and improving the abundances of *Lachnospiraceae* and *Rikenellaceae.* Many enteric pathogens can use the sugars in the intestinal mucus as food for growth. Therefore, the use of beneficial bacteria occupying the same ecological niche (bacteria able to utilize mucin monosaccharides) to crowd out the living space of pathogenic bacteria is a potential microecological treatment strategy [43]. Mucin monosaccharides can be utilized by some members of the *Lachnospiraceae* and *Rikenellaceae* families. In addition, *Lachnospiraceae* participates in producing short-chain fatty acids and maintaining intestinal immune homeostasis [44,45]. Therefore, *B. lactis* SF may restore the balance of intestinal flora by increasing the relative abundance of bacteria that can occupy the same ecological niche of pathogenic bacteria, thus reducing the living space of pathogenic bacteria.

When the gut microbiota is disrupted, the concentration of microbiota products such as LPS increases, which binds to Toll-like receptors on the cell surface, activates NF-κB in the cytoplasm, produces and secretes inflammatory cytokines and chemokines that disrupt tight junctions in the intestinal epithelium, and downregulates intestinal barrier function. The breakdown of the intestinal barrier can lead to the influx of many toxic factors from the gut into the liver through the portal vein, causing liver inflammation [46]. The NAFLD model diet impaired intestinal barriers and significantly downregulated the expression of TJs in mice, while the *B. lactis* SF intervention significantly alleviated symptoms. The NAFLD model diet led to a significant increase in serum LPS content, which in turn led to upregulated expression levels of TLR4 receptor, IKK-β, IκB-α, NF-κB, TNF-α, IL-6, and other inflammatory cytokines, which were significantly alleviated by *B. lactis* SF intervention. The results indicate that *B. lactis* SF can protect the intestinal barrier function by regulating the structure of intestinal flora, thus inhibiting TLR4/NF-κB signaling and the secretion of inflammatory cytokines and alleviating liver injury.

NAFLD is often associated with features of insulin resistance [47]. The accumulation of hepatic lipids reduces the sensitivity of the liver to insulin, thus increasing serum glucose levels. Subsequently, the insulin levels increase [48]. Insulin resistance can be considered as a chronic inflammatory disease. TNF-α and IL-6 are considered the main inflammatory mediators of insulin resistance, which can activate various inflammatory signaling pathways, inhibit insulin signaling, and lead to insulin resistance [49,50]. Under normal conditions, insulin stimulates the tyrosine kinase activity of the insulin receptor, and the binding of insulin to its receptor initiates the IRS-1activation, which regulates the phosphorylation of PI3K 3, and thus activates the downstream Akt phosphate [51]. mTOR is a downstream target of the PI3K-Akt pathway, which can regulate lipid synthesis through SREBP-1c and is closely related to lipid metabolism [52,53]. SREBP-1c is a major regulator of hepatic fatty acid synthesis [54], and it can control the content of fatty acids and their metabolites in the body by regulating ACC1 and other enzymes or proteins involved in fatty acid synthesis [55]. The AMPK pathway is a classical pathway for regulating lipid metabolism. Activated AMPK can inhibit the expression of SREBP-1c and C/EBPα to downregulate the expression of lipid production-related proteins and reduce fatty acid production in the liver. AMPK can promote fatty acid oxidation by activating PPAR-γ, thereby reducing lipid accumulation in vivo [56,57,58]. Impaired adipocyte function and the production of inflammatory mediators such as TNF-α and IL-6 can reduce the expression of PPAR-γ [59]. Results showed that *B. lactis* SF could alleviate HFD-induced insulin resistance, maintain the balance of lipid metabolism in the liver by regulating mRNA expression of genes associated with the PI3K-Akt/AMPK signaling pathway, and reduce the accumulation of fat in the liver, thereby alleviating NAFLD. This finding was obtained possibly because *B. lactis* SF can relieve inflammation and reduce the release of inflammatory factors. However, the expression of proteins associated with this pathway has not been validated in this study. This mechanism could be further studied in depth in the future.

Oxidative stress plays an important role in the development of NAFLD. In the context of NAFLD [60,61], liver lipid accumulation can lead to the overproduction of reactive oxygen species, leading to oxidative stress. This condition is mainly manifested as an increase in the superoxide product MDA and a decrease in the antioxidant enzymes SOD and CAT [61,62,63]. SOD and CAT are the main substances for scavenging free radicals in organisms and are the first line of defense against oxidative stress [64,65]. MDA is a product of unsaturated lipid peroxidation and is considered a toxic molecule and biomarker of oxidative stress. MDA content can reflect the lipid peroxidation rate and intensity of the body, which indirectly reflects the degree of tissue peroxidative damage [66]. In addition, when hepatocytes are damaged, the intracellular GSH content is reduced, various GSH-dependent enzymes are inactivated, and the protective effect on oxidative free radicals is weakened. In the present study, compared with the HFD group, the hepatic SOD and CAT activities and GSH content in the SF group remarkably increased, and the MDA content significantly decreased, indicating that *B. lactis* SF significantly alleviated the oxidative stress caused by the NAFLD model diet. Therefore, *B. lactis* SF showed obvious antioxidant effects in vivo, which is consistent with the results of in vitro experiments. The reactive oxygen species generated during oxidative stress can induce autophagy, which plays an important role in regulating cell survival/death. Moreover, autophagy can aggravate liver damage. Beclin1 is a protein that is involved in the regulation of autophagy, and microtubule-associated protein light chain 3 (LC3) has been widely used as a marker of autophagy [67]. The content of LC3II in the liver tissues of mice in the HFD group increased, whereas that in the SF group was significantly lower than that in the HFD group, indicating that *B. lactis* SF can inhibit autophagy.

## 5. Conclusions

*B. lactis* SF has excellent probiotic properties including tolerance to gastrointestinal fluids and excellent intestinal colonization as well as antibacterial and antioxidant capacities. In vivo, *B. lactis* SF inhibited the TLR4/NF-κB signaling pathway by regulating the intestinal flora and repairing the intestinal barrier, thereby reducing the production of inflammatory factors. In addition, *B. lactis* SF alleviated inflammation along with insulin resistance, thereby regulating the mRNA expression of genes associated with the PI3K-Akt/AMPK signaling pathway and reducing hepatic fat accumulation. *B. lactis* SF attenuated oxidative stress and further alleviated autophagy. In summary, this study provides candidate strains for the development of probiotics that can alleviate and treat NAFLD.

## Figures and Tables

**Table 1:** Antibiotic susceptibility of *B. lactis* SF.

Class	Antibiotic	Content (μg)	Diameter of Inhibition Zone (mm)		Sensitivity ^1^	
			LGG	SF	LGG	SF
Aminoglycoside	Gentamicin	10	13	13	I	I
	Streptomycin	10	10	9	R	R
	Kanamycin	30	8	6	R	R
Glycopeptide	Bacitracin	0.04U	6	6	R	R
	Polymyxin B	30	6	6	R	R
	Vancomycin	30	6	6	R	R
Quinolones	Ciprofloxacin	5	14	6	I	R
Beta-lactams	Amoxicillin	10	12	10	R	R
	Ampicillin	10	13	15	I	I
Tetracyclines	Tetracycline	30	22	17	S	S
Cephalosporins	Cefoxitin	30	6	6	R	R
Amphenicols	Chloramphenicol	30	22	20	S	S
Macrolide	Erythromycin	15	21	21	S	S
Coumarins	novobiocin	5	17	15	I	I
Sulfonamides	Rifampicin	5	23	17	S	I
	Sulphamethoxazole	25	6	26	R	S

^1^ S = susceptible; I = intermediate; R = resistance.
